# Anti-inflammatory effects of *Lactobacillus casei *BL23 producing or not a manganese-dependant catalase on DSS-induced colitis in mice

**DOI:** 10.1186/1475-2859-6-22

**Published:** 2007-07-20

**Authors:** Tatiana Rochat, Luis Bermúdez-Humarán, Jean-Jacques Gratadoux, Christel Fourage, Christine Hoebler, Gérard Corthier, Philippe Langella

**Affiliations:** 1 Unité d'Ecologie et Physiologie du Système Digestif, Centre de Recherche INRA, Domaine de Vilvert, 78352 Jouy-en-Josas cedex, France; 2 Physiologie Intestinale, Croissance et Nutrition Humaine, UMR INRA/Université de Nantes, Rue de la Géraudière – BP 71627, 44316 Nantes cedex 3, France

## Abstract

**Background:**

Human immune cells generate large amounts of reactive oxygen species (ROS) throughout the respiratory burst that occurs during inflammation. In inflammatory bowel diseases, a sustained and abnormal activation of the immune system results in oxidative stress in the digestive tract and in a loss of intestinal homeostasis. We previously showed that the heterologous production of the *Lactobacillus plantarum *ATCC14431 manganese-dependant catalase (MnKat) in *Lb. casei *BL23 successfully enhances its survival when exposed to oxidative stress. In this study, we evaluated the preventive effects of this antioxidative *Lb. casei *strain in a murine model of dextran sodium sulfate (DSS)-induced moderate colitis.

**Results:**

Either *Lb. casei *BL23 MnKat^- ^or MnKat^+ ^was administered daily to mice treated with DSS for 10 days. In contrast to control mice treated with PBS for which DSS induced bleeding diarrhea and mucosal lesions, mice treated with both *Lb. casei *strains presented a significant (*p *< 0.05) reduction of caecal and colonic inflammatory scores.

**Conclusion:**

No contribution of MnKat to the protective effect from epithelial damage has been observed in the tested conditions. In contrast, these results confirm the high interest of *Lb. casei *as an anti-inflammatory probiotic strain.

## Background

Inflammatory bowel diseases (IBD), including Crohn's disease and ulcerative colitis, are characterised by an abnormal activation of the gut-associated immune system resulting in a chronic inflammation of the digestive tract (DT). IBD patients show flares of remission and relapses with symptoms of bloody diarrhea, abdominal pain and rectal bleeding. Although the etiology of IBD remains unclear, it is generally considered that a combination of several factors including genetic predisposition, immune disorders and environmental factors could be involved. The role of commensal and pathogenic bacteria in the induction of these diseases is particularly well documented [1-7].

Recent studies linked intestinal oxidative stress to the observed epithelial damage. In small quantities, reactive oxygen species (ROS) are involved in certain signalization or regulation pathways. During normal inflammation, ROS toxicity leads to the elimination of infectious agents: ROS are generated inside the DT during oxidative burst by activated phagocyte cells which possess ROS-producing enzymes, such as NADPH oxidase [8], NO synthase [9] and myeloperoxidase [10] and they infiltrate the lamina propria. However, in the case of IBD, excessive amounts of ROS accumulate and lead to oxidative epithelial damage. Several studies have shown a correlation between the increase in ROS production and disease activity in inflamed biopsies of IBD patients [11-14]. The measured effects on antioxidative systems diverge between studies: antioxidative enzyme activity was either increased [15,16] or decreased [12] in IBD biopsies, depending on the nature of the enzyme and the state of disease activity. Moreover, several studies provide direct evidence of *in vivo *oxidative injury in inflamed epithelial cells of IBD patients [12,17,18]. Recently, several antioxidative strategies have been evaluated using animal colitis models and appear to be efficient in the reduction of inflammatory damage [19-23]. These antioxidative strategies could be based on the activity of two enzymes: superoxide dismutase (SOD) which reduced superoxides and catalases which catabolized in H_2_O + O_2 _the hydrogen peroxide resulting from the reduction of superoxides. Here, we proposed to evaluate the efficiency of *in situ *delivery of one catalase by the lactic acid bacterium (LAB) *Lactobacillus casei*. In parallel to their traditional use for the production of fermented products, LAB are also studied for their probiotic properties. Some strains could improve IBD patients' health, as it observed, for example, with the administration of the VSL#3 probiotic cocktail which delayed the relapse into pouchitis after surgical resection [24,25]. Natural anti-inflammatory effects were recently shown for *Lb. salivarius *[26], *Bifidobacterium *and *Lb. plantarum *[27,28] and *Lb. casei *Shirota [29,30] using experimental colitis models. New recombinant strategies are also in progress to engineer LAB strains for *in situ *delivery of heterologous therapeutic proteins. This strategy has been first applied to IBD, where the intragastric administration of a genetically engineered *L. lactis *strain producing an anti-inflammatory cytokine, the interleukin-10 (IL-10), caused a significant reduction in colitis in mice treated with DSS [31].

We previously showed that the improvement of the antioxidative capacities of *Lactococcus lactis *and *Lb. casei *by the introduction of a catalase gene led to an increase of bacterial survival in the presence of hydrogen peroxide and under aerated conditions. We evaluated the *in vitro *catalase activity of *Lb. casei *BL23 producing the manganese-dependant catalase (hereafter called MnKat) from *Lb. plantarum *ATCC14431 [32] at 16 μmol of H_2_O_2_/min/mg of protein [33,34]. In this study, the preventive effects of two isogenic *Lb. casei *strains producing or not MnKat on the development of the inflammation were analyzed using a DSS-induced intestinal colitis model in BALB/c mice. We tested *in vivo *whether the MnKat-producing *Lb. casei *strain could reduce epithelial damage of the digestive tract.

## Results

### Characterization of DSS-induced colitis

**Figure 1:**
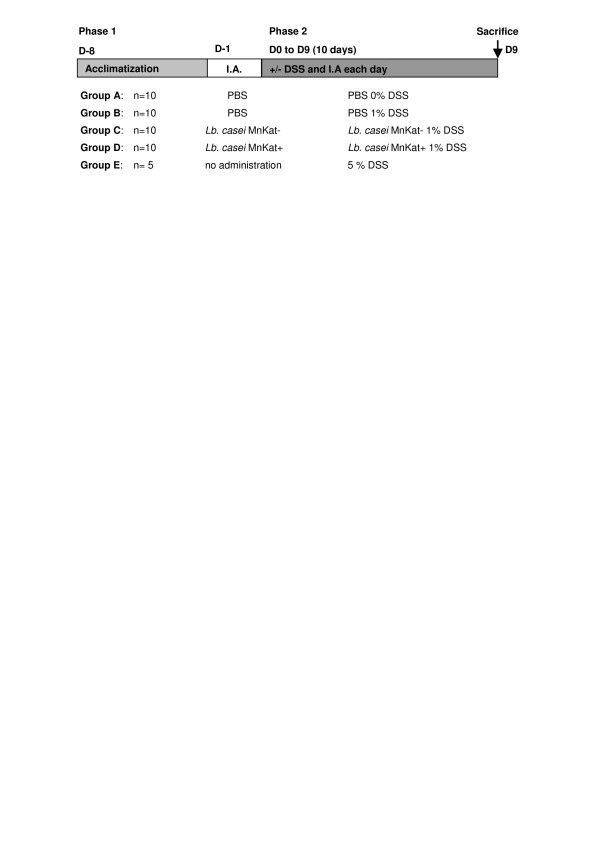
**Experimental protocol for the induction of moderate colitis**. Phases 1 and 2 correspond to mice acclimatization and to colitis induction (DSS addition in drinking water from J0), respectively. « I.A. » means « intragastric administration ». Groups of BALB/c mice were used to evaluate the effects of different treatments. Groups used as controls were: i) treated with PBS and either DSS 0% (no inflammation control) or DSS 1% (inflammation control) or ii) treated with *Lb. casei *MnKat – with DSS 1% (control used to dissociate the effects due to the LAB and those due to MnKat). Group E represents a severe colitis control (5% DSS).

**Figure 2:**
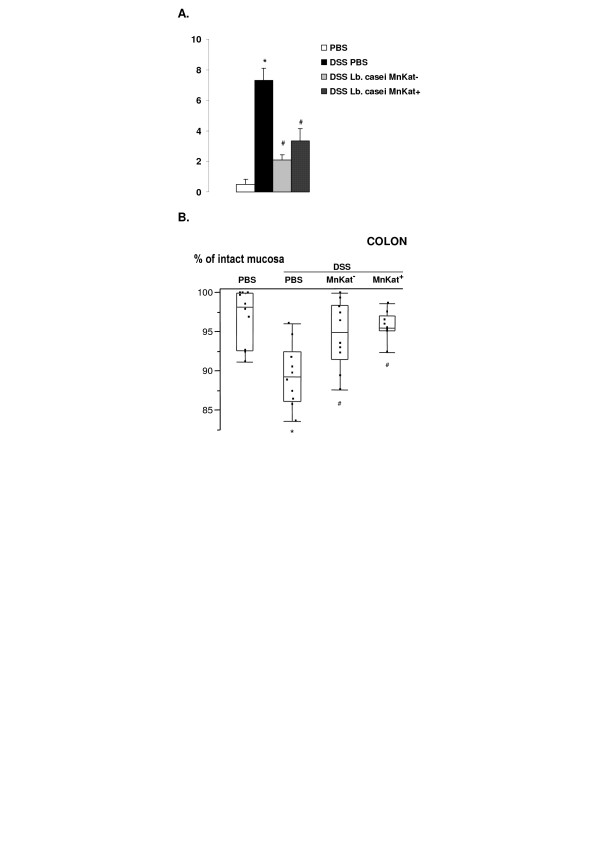
**Effects of *Lb. casei *BL23 on the inflammation**. **A. Total macroscopic score**. Total macroscopic score was the sum of caecal and colonic scores. Data are expressed as mean ± SEM, *n *= 10/treatment. (*) P < 0.05 *versus *no inflammation group (PBS) and ^(#) ^*versus *inflamed control group (PBS DSS). **B. Histological score**. This parameter is expressed as percentage of intact mucosa and presented using box-and-whisker plots, *n *= 10/treatment. (*) P < 0.05 *versus *no inflamed group (PBS) and ^(#) ^*versus *inflamed control group (PBS DSS).

We first analyzed the effect of 1% DSS administration to mice for 10 days on clinical symptoms and inflammatory scores using the experimental design described in Figure 1. After 10 days of DSS administration, no significant differences in decrease of body weight, food and water intake, were observed between group B (1% DSS) and group A (0% DSS) (data not shown). Total stool score (based on consistency of stool and bleeding) was significantly increased in group B when compared to the untreated group A (10 *versus *~0; data not shown), with bleeding stools for all mice of group B during the experiment. The macroscopic score (mucosal thickening, hyperaemia, ulcerations) was also significantly increased in DSS-treated group (Figure 2A). The percentage of intact mucosa, taking into account shortening of the crypts and inflammatory infiltrates, decreased significantly showing histological damages (Figure 2B). Ours results showed that i) inflammatory symptoms induced by our experimental protocol are significantly different between treated and untreated groups and ii) intestinal inflammation corresponds to moderate colitis. In parallel, a more drastic treatment (5% DSS for 10 days; group E) was tested as a severe colitis control group. In this case, we observed i) a decrease (15%) of body weight compared to untreated mice, ii) a reduction (~30%) of water and food intake and iii) a total stool score two-fold higher than after 1% DSS treatment (21 *versus *10 respectively). Moreover, a significant shortening of colonic length (40%) and absence of digestive contents replaced by severe bleeding were observed in sacrificed mice. Considering the severity of the symptoms, we thought that 5% DSS was too high to detect any positive effect of the bacterial treatment.

**Figure 3:**
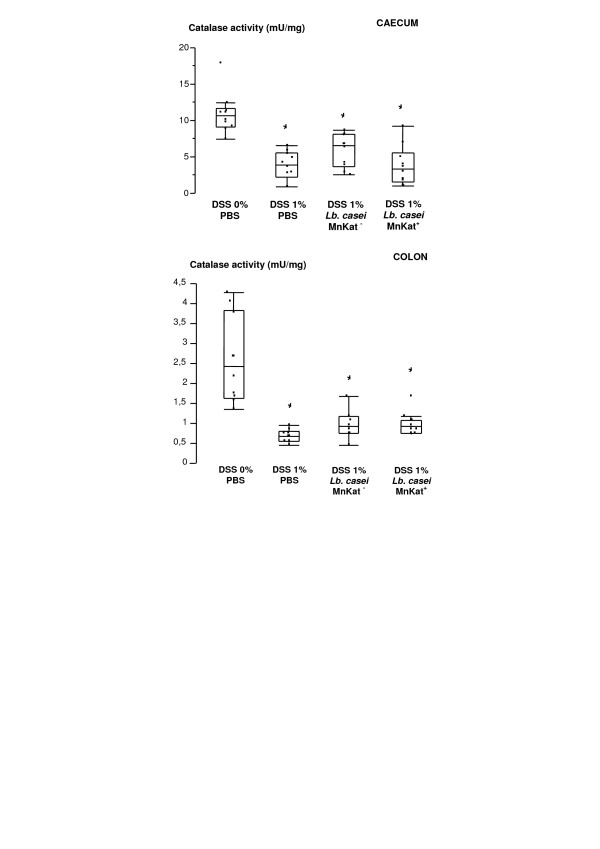
**Catalase activity in digestive contents of DSS-induced colitis in mice**. Digestive contents homogenates were prepared from MnKat^+ ^treated mice and from control groups (PBS 0% DSS, PBS 1% DSS and MnKat^- ^1% DSS). Total proteins were assayed using Bradford method. Total catalase activity was assessed using colorimetric method and expressed as mU/mg of total protein using box-and-whisker plots, *n *= 10/treatment. (*) P < 0.05 *versus *no inflamed group (PBS).

Nevertheless, a significant decrease of total catalase activity was observed in digestive contents of 1% DSS-treated mice (Figure 3) and this led us to adopt the 1% DSS model for further experiments to test the antioxidative potential of our strains.

### Effects of *Lb. casei *BL23 producing MnKat on intestinal inflammation

To evaluate the effects of *Lb. casei *BL23 producing or not MnKat catalase on inflammation development, two groups of mice received 5 × 10^9 ^cfu/mouse of either *Lb. casei *MnKat^- ^or *Lb. casei *MnKat^+ ^by intragastric administration one day before starting DSS administration and then daily until sacrifice. Clinical symptoms were compared with DSS-treated mice receiving PBS. As noted above, DSS treatment did not induce body weight or food intake differences compared to untreated mice (group A). *Lb. casei *BL23 administration did not modify these parameters. Total stool scores were similar for each treated group (10.5; 8.6 and 9.9 for PBS, *Lb. casei *MnKat^- ^and MnKat^+ ^groups, respectively) confirming the action of DSS. This parameter showed that either *Lb. casei *BL23 MnKat^+ ^or MnKat^- ^administration failed to prevent totally the development of intestinal inflammation. However, inflammatory scores (macroscopic and histological ones) showed that *Lb. casei *BL23 administration limits intestinal mucosal and epithelial damages: i) Total macroscopic scores (Figure 2A) are significantly reduced from ~7 to ~2 and ~3 for PBS and *Lb. casei *MnKat^- ^or MnKat^+ ^DSS-treated groups, respectively; and ii) Histological scores were significantly different between DSS-treated and untreated PBS-control groups: The percentage of intact mucosa decreased from 97% to 88% in colonic mucosa for DSS untreated and treated groups, respectively (Figure 2B). Administration of *Lb. casei *BL23 led in the colon to significant reduction of inflamed epithelial damage. *Lb. casei*-treated groups are not significantly different from both DSS untreated and treated groups (data not shown). Histological scores of *Lb. casei *BL23 DSS-treated groups (expressed as percentage of intact mucosa) are not significantly different from DSS-untreated group, in caecum and colon (Figure 2B). No significant difference was observed between MnKat^+ ^and MnKat^-^-treated mice for both macroscopic and histological scores.

We then analysed the effects of both DSS treatment and *Lb. casei *BL23 administration on mucosal immune cells by ELISA assays. TNF-α and IFN-γ pro-inflammatory cytokines, IL-10 anti-inflammatory cytokine and IL-4 Th2-cytokine were quantified in samples of digestive tissues. Contrary to macroscopic and histological scores, no significant difference of cytokines level was observed between DSS-treated and untreated groups and between *Lb. casei *BL23 MnKat^+ ^or MnKat^- ^treated mice (data not shown).

To estimate the level of oxidative stress induced in our experimental protocol, a quantification of lipid peroxidation in both caecal and colonic tissue homogenates was performed using a malondialdehydes (MDA) assay. MDA concentration was standardised using a commercial MDA solution. The statistical analysis of MDA levels revealed no significant difference between DSS-treated and untreated PBS-control groups (data not shown).

Twenty-four hours after the intragastric administration of 5.10^9 ^cfu/mouse, the number of viable cells of *Lb. casei *BL23 MnKat^+ ^or MnKat^- ^in caecal, proximal and distal colonic contents, was determined: The level of *Lb. casei *BL23 was ~8.10^5 ^cfu/g of digestive contents, in the three tested compartments. The presence of MnKat had no effect on *Lb. casei *BL23 survival in digestive contents.

The total catalase activity in digestive contents from caecum and colon of DSS-treated and untreated mice and of *Lb. casei *MnKat^- ^and MnKat^+^-treated mice was quantified. A significant decrease was observed for all DSS-treated groups compared to untreated mice for both caecal and colonic homogenates (Figure 3). No significant difference was observed between PBS and *Lb. casei *MnKat^+ ^or MnKat^-^. The production of MnKat by *Lb. casei *BL 23 did not restore the initial level of catalase activity in digestive contents (Figure 3).

In summary, our results showed that the intragastric administration of *Lb. casei *BL23 limits but does not impair the development of mucosal and epithelial inflammation damage in both caecum and colon of DSS-treated mice. This suggests that i) 1% DSS model leads to a significant decrease of catalase activity in digestive contents; ii) *Lb. casei *BL23 exerts natural anti-inflammatory effects by still unknown mechanisms and that iii) the delivery of MnKat catalase has no effect on the development of inflammation in the tested conditions.

## Discussion and conclusion

The aim of this study was to determine the impact of the *in situ *delivery of catalase-producing lactobacilli in the DT on the development of inflammation and oxidative intestinal damage in mice. Our strategy, based on the intragastric administration of both isogenic strains of *Lb. casei *BL23 producing or not MnKat, allowed us to distinguish the respective effects of the strain and of the catalase activity. Both *Lb. casei *BL23 treatments led to anti-inflammatory effects for DSS-treated mice, suggesting that the BL23 strain itself is responsible for the protective effect. The mechanisms and bacterial components involved in these anti-inflammatory effects remain unknown.

In the tested conditions, no protection due to the catalase was observed. Two hypotheses could explain this result: i) no induction of effective oxidative stress in our moderate colitis model and/or ii) a too low catalase activity delivered in digestive tractus via *Lb. casei *BL23 MnKat^+^. No increase of intestinal oxidative stress in our model was detected using a first method (evaluation of MDA level in digestive tissues homogenates).

Using a colorimetric method, we observed for the first time a significant decrease of the total catalase activity in the intestinal lumen of all DSS-treated mice. The four-fold higher catalase activity of caecal contents compared to colonic contents is coherent with the decrease of aerobiosis from the stomach to the rectum. DSS-induced modifications of the homeostasis of the intestinal microbiota could explain this reduction of catalase activity. Dominant colon species are strict anaerobic bacteria like *Bacteroides*, *Bifidobacterium, Clostridium coccoides *and *Cl. leptum *whereas other groups like *Enterobacteriaceae *or *Lactobacillus *are subdominant. Among microbiota, some bacteria possess catalase activity as described for *Bacteroides fragilis *[35]. A loss of diversity of *Bacteroides *and *Enterobacteriaceae *groups was observed in biopsies of patients with Crohn's disease or ulcerative colitis [4]. In our experiments, the reason of the observed decrease is difficult to determine because the composition of the microbiota of the conventional mice used is unknown. Previous studies showed that activities of antioxidative enzymes produced by epithelial cells were modified in biopsies of patients with Crohn's disease or ulcerative colitis [12,15,16]. In this study, we showed for the first time a reduction of antioxidative defences of caecal and colonic contents during the induction of inflammation. All these data suggest a global deficiency of antioxidative enzymes during intestinal inflammation that might be involved in the mechanism of development of the disease.

The second reason of the absence of any incidence of MnKat production could be a too low catalase activity and more generally, a too low antioxidative potential. Our results showed that the administration of our MnKat-producing strain did not restore the initial catalase activity level in the digestive contents. Increasing catalase activity seems to be necessary. To optimise our antioxidative strategy, evaluation of the effects of co-administration of *Lb. casei *BL23 strains producing high levels of MnKat and SOD from *L. lactis *[36] as some previous studies showed the positive impact of increased SOD activity in intestinal inflammation models will be relevant [20,21,37,38].

In our test conditions, no increase of mucosal pro-inflammatory cytokines (TNF-α and IFN-γ) or decrease of IL-4 and IL-10 was observed in DSS-treated mice. The administration of both *Lb. casei *BL23 strains did not induce modification of mucosal cytokines. In contrast, an increase of TNF-α in DSS-induced colitis was previously observed either at the gene expression level [39,40] or at the systemic protein level [22,41]. In the DSS-colitis model, the modification of mucosal cytokine levels could occur with the apparition of chronic inflammation depending of the mice used: Melgar *et al*. (2005) observed an increase of IL-1β, IL-12 and IFN-γ levels between 100- and 1000-fold in C57Bl/6 mice (where the colitis progresses in chronicity) in contrast to BALB/c mice where cytokines levels did not increase [42]. These data could explain the absence of the modulation of cytokine levels in our experiments performed with BALB/c mice. For now, the pathways responsible for inflammation development in the DSS-colitis model are not clearly understood. No modulation of mucosal cytokines by *Lb. casei *BL23 administration *in vivo *was observed. So far, such modulations were only observed in *in vitro *co-cultures between LAB and peripheral blood mononuclear cells [27,30]. No published data correlate cytokine modulations by LAB in *in vitro *and *in vivo *assays.

In conclusion, our results showed anti-inflammatory capacities of *Lb. casei *BL23 strain in a DSS-induced colitis model. This original result is in accordance with a recent one observed in TNBS-induced colitis [43] and previous ones obtained with other *Lb. casei *strains. Both Shirota and DN-114001 strains impair inflammation development in a DSS-induced [29,30] and TNBS-induced [44] colitis model, respectively. These two colitis models mimic Crohn's disease (for TNBS) and ulcerative colitis (for DSS) and the efficiency of these *Lb. casei *strains in both open interesting potential uses of them. Several studies based on host-probiotic interactions aimed to identify the still unkown mechanisms responsible for these anti-inflammatory effects: Borruel *et al. *first observed that *Lb. casei *and *Lb. bulgaricus *are able to interact with immune cells and to modulate pro-inflammatory cytokines using co-cultures of LAB with biopsies of patients with active Crohn's disease, but the mechanism of action is still not known [45]. Two recent studies established a link between probiotic anti-inflammatory capacities and Toll-like receptors (TLR) pathway [27,46]. Although the involvement of TLR in the stimulation of the immune system by the bacterial components is well documented, their implication in inflammatory diseases remains confusing: they were also shown to be essential to maintain a mucosal homeostasis during intestinal inflammation, as observed with MyD88^-/- ^knockout mice which are very sensible to the induction of DSS colitis [5].

The increasing availability of probiotic genomic sequences constitutes a major progress to understand molecular mechanisms involved in the anti-inflammatory capacities observed. This will be facilitated by the use in parallel of cellular and animals models and could result in a global view of probiotic-host interactions.

## Methods

### Animals and treatments

Conventional male BALB/c mice (Janvier, Le Genest Saint Isle, France), 7 wk of age, were reared by groups of 5 mice per cage in Texler-type isolators (La Calhène, Vélizy, France) to limit GMO dissemination, and maintained in an environmentally controlled room (21°C) with a 12-h light-dark cycle as described [47]. Food (UAR, Villemoisson, France) and water were consumed *ad libitum*; food intake and body weight were recorded every day. All experiments were carried out in accordance with the institutional guidelines.

### Bacterial strains and growth conditions

Cultures of both strains of *Lb. casei *BL23 containing either the control plasmid pLEM415 (« MnKat^- ^») or catalase-carrier plasmid pLEM415*mnkat *(« MnKat^+ ^») were performed in MRS medium (Difco) containing erythromycin (Em; 5 μg/mL) and incubated 24 h at 37°C. The bacterial suspension used for the treatment of mice was prepared as follows: 50 mL of stationary phase cultures were centrifuged (6000 g, 10 min), and the collected cells were washed and resuspended in phosphate buffered saline (PBS; NaCl 8 g/L, KCl 0.2 g/L, NaH_2_PO_4 _1.44 g/L, K_2_HPO_4_0.24 g/L pH 7.4) to obtain a final concentration of 2.5.10^10 ^cfu/mL (25-fold concentrated cultures). Bacterial population level of cell suspensions was checked by cfu counting, and catalase activity was verified as previously described [34]. Briefly, 10 μL of H_2_O_2 _(8 M) was added to 30 μL of bacterial suspension and O_2 _gas bubbles coming from H_2_O_2 _degradation were observed. Each mouse received 0.2 mL of bacterial suspension by intragastric administration (5.10^9 ^cfu/mouse/day). For enumeration of *Lb. casei *in the DT, digestive contents were dissolved in cold peptone (1 g/L; dilution 1/10 = 1 mL for 0.1 g of digestive contents) and adequate dilutions were plated on MRS containing Em (5 μg/mL) and nalidixic acid (40 μg/mL), an inhibitor of Gram negative bacteria, using a SpiralPlater^® ^(Interscience, France) and incubated 48 h at 37°C before counting.

### Induction of colitis

The experimental protocol is presented in Figure 1. The main experiment was performed with 4 groups of mice (n = 10). After acclimatization, colonic inflammation was induced by the addition of 1% (w/v) DSS (40 kDa; ICN n°160110) in drinking water for 10 days (group B/C/D; DSS^+^) until their sacrifice for digestive tissue collection. The no-inflammation control group received no DSS (group A; DSS^-^). An additional group (E; n = 5), was used as severe colitis control and received 5% DSS. Intragastric administration of bacteria was performed the day before the beginning of DSS treatment and each following day until sacrifice: groups A and B received PBS, group C *Lb. casei *MnKat^- ^and group D *Lb. casei *MnKat^+^.

### Collection of digestive tissues and contents

**Figure 4:**
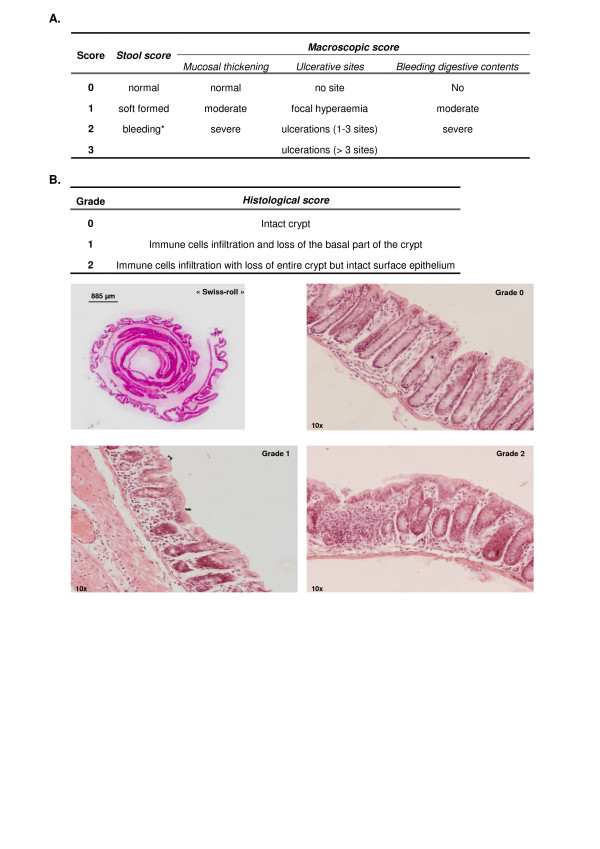
**Inflammation scores determination**. **A. *Stool and macroscopic scores***. Stool scores were determined during the phase of colitis induction, taking into account stool consistency and bleeding. The three macroscopic parameters (mucosal thickening, ulcerative sites and bleeding inside digestive contents) were added together to obtain macroscopic scores for caecum, proximal and distal colon. Separate scores were added up to obtain a total macroscopic score for each mouse. (*: presence of blood using HémocultII^® ^detection test). **B. *Histological score***. Swiss rolls were used to calculate a percentage of intact mucosa for each digestive segment. Three grades were defined depending on the mucosal state. The percentage of mucosal length in grade 0, 1 or 2 was calculated by microscopic observation of hematoxylin/eosin-stained sections (5 μm) and using LuciaG^® ^software to measure the length of each section. Results were expressed as percentage of intact mucosa.

After the 10 days of DSS treatment, the animals were killed by cervical dislocation. Three segments of the digestive tract (caecum, proximal colon and distal colon) were then removed; caecal and colonic contents were aseptically collected and immediately used for *Lb. casei *enumeration (group C and D; n = 10) or for total catalase activity assessment (n = 10). Caecal, proximal and distal colonic mucosa were cut longitudinally and cleaned with sterile PBS. After evaluation of macroscopic damage and for each DT segment, i) for histological examination, mucosa was rolled up on itself over the full length to obtain a "swiss roll" (Figure 4B) and immediately fixed in 4% formaldehyde PBS pH 7.4, dehydrated, and paraffin embedded for histological observations (n = 10) or ii) for MDA assay, mucosa was immediately placed in Tris-HCl buffer (0.1 M, pH 7.5, Tween 80 0.1% ; 60 mg of digestive tissues/mL) before homogenisation and iii) for cytokines assays, a digestive tissue segment (0.5 cm) was placed in 500 μL of cold antiprotease buffer (Protease Inhibitor Cocktail Tablets, Roche^®^) before homogenisation using a BeatBeater™ (BioSpec Products). A second lot (four groups of 10 mice) which received the same experimental design was used to assay lipid peroxidation (see the corresponding paragraph).

### Assessment of colonic damage and inflammation

#### Body weight and stool score

Clinical symptoms were assessed every day during the course of the experiment. Body weight gain and stool scores were calculated until sacrifice following the protocol of Gaudier *et al *[39] with slight modifications: A stool score (Figure 4A) was calculated each day taking account of consistency and bleeding. The presence of digestive contents and faecal blood was assessed visually and with HémocultII^® ^detection test (SKD, France). Total stool score was the sum of daily scores (from D0 to D9) for each mouse.

#### Macroscopic and histological scoring of mucosa inflammation

Macroscopic damage of caecal and colonic mucosa was scored visually using a scale taking into account three parameters (mucosa thickening, ulceration and bleeding in contents) as described previously by Gaudier *et al *[39] (Figure 4A). Caecal and colonic macroscopic scores were obtained by addition of these three partial scores. Total macroscopic scores were the sum of caecal and colonic scores. For histological observations, sections (5 μm) of paraffin embedded tissues were stained with haematoxylin and eosin, and observed by microscopy for inflammation scoring. Histological crypt scoring for inflammation was performed blindly and took into account the extent of immune cells infiltration, integrity of the crypts and of surface epithelium (Figure 4B). The proportion of mucosa length area involved for each grade (0 to 2) was quantified by image analysis with LuciaG^® ^software (Laboratory Imaging Ltd, Praha, Czech Republic). Percentage of intact mucosa area [Σ(area in grade 0)/Σ(area in grade 0, 1 and 2) × 100] was calculated for each DT segment.

### Cytokine assays

Digestive tissues of caecum, proximal and distal colon were mechanically homogenized in cold antiprotease buffer using a BeatBeater™ (BioSpec Products) and after centrifugation for 30 min at 16000 g and 4°C, the supernatant was stocked at -20°C. Total proteins were quantified in digestive homogenates by a Bradford assay. A mix of the three homogenates (caecum, proximal and distal colon) in equal proportion of total proteins was prepared for each mouse and used for the assay of cytokines (TNFα, IFNγ, IL-10 and IL-4) using ELISA kits (Ebioscience^®^). Cytokine levels are expressed in ng/mg of total proteins.

### Lipid peroxidation

An estimation of the level of lipid peroxidation in digestive tissues was obtained using the method described by Gérard-Monnier *et al *[48] which is based on the colorimetric assay of malondialdehydes (MDA, derived product of peroxidized lipids). Digestive tissue homogenates (divided in two parts: caecum and colon) were obtained by mechanic disruption using a BeatBeater™ (BioSpec Products). After centrifugation at 16000 g for 15 min, 4°C, supernatants were stocked at -80°C. For the assay, 75 μL of homogenate were acidified by the addition of 75 μL of sodium acetate buffer (0.4 M) containing 5% concentrated HCl (pH ~1.2) and hydrolyzed for 80 min at 60°C. After cooling, 700 μL of 1-methyl-2-phenylindole (14.3 mM; dissolved in acetonitrile/methanol 3:1) and 150 μL of concentrated HCl were added. The mixture was incubated at 45°C for 80 min. Produced MDA forms a stable chromophore which absorbs light at 586 nm (absorbance measured using a spectrophotometer after centrifugation at 8000 g 10 min). Non-specific coloration was measured using homogenate buffer instead of tissue homogenate. The reaction was standardised with commercial hydrolysed MDA (Merck, Darmstadt, Germany) and the results expressed in nmol of MDA/g of tissue.

### Catalase activity assay in digestive contents

The assay was performed using the Amplex-Red^® ^kit (Invitrogen). Caecal and colonic contents were collected and suspended in 500 μL of cold reaction buffer supplemented with antiprotease (Protease Inhibitor Cocktail Tablets, Roche^®^). Homogenates were obtained by mechanical disruption for 40 s at speed 6.5 using a Fast Prep FP120 (ThermoSavant). After centrifugation (15 min, 16000 g, 4°C), supernatants were aliquoted and stocked at -80°C. Total proteins were quantified in the extracts using the Bradford method. Calorimetrically assayed, catalase activity was standardised using a commercial catalase solution and expressed in mU/mg of total protein.

### Statistical analysis

All data are expressed as mean ± SEM. The effects of DSS treatment and *Lb. casei *administration were assessed by an ANOVA test using JUMP5.1^® ^software. Differences with P < 0.05 were considered significant.

## Authors' contributions

TR, JJG, LGBH, CH and CF carried out the molecular genetic studies and participated in experiments. TR and PL drafted the manuscript. LGBH carried out the immunoassays. CH and GC participated in the design of the study. TR and LGBH performed the statistical analysis. All authors read and approved the final manuscript.
